# 45 Juvenile systemic lupus erythematosus: about a series of 35 cases

**DOI:** 10.1093/rheumatology/keac496.041

**Published:** 2022-10-07

**Authors:** R El Qadiry, H Nassih, A Bourrahouat, I Ait Sab

**Affiliations:** Pediatric B Department—Mother-Child Pole—Mohammed VI University Hospital, Marrakesh

## Abstract

**Background:**

Systemic Lupus Erythematosus (SLE) is a chronic autoimmune disease that can involve any organ system with a wide range of disease manifestations, and can lead to significant morbidity and even mortality.

**Objectives:**

To describe the epidemiology, common clinical features, complications, and management of SLE in children.

**Methods:**

A single center retrospective descriptive study of 35 children with SLE.

**Results:**

There were 27 (77%) girls and 8 (22.9%) boys. The mean age at diagnosis was 9 ± 2 years, with extremes of 3 and 15 years. Systemic involvement was in the form of protracted fever in 62.8%, nephrotic syndrome in 37%, nephritic syndrome in 40%, thrombotic microangiopathy in 8.6%, isolated proteinuria in 25.7%, acute kidney injury in 25.7%, malar rash in 48.6%, arthritis in 57%, neurolupus in 17%, serositis in 11.4%, and hemolytic anaemia in 28.6%. Coexisting autoimmune conditions were systemic sclerosis, antiphospholipid antibodies syndrome and Gujerat-Sjogren syndrome. Two cases had severe pancreatitis as a revealing pattern. Posterior uveitis was present in one case. Meanwhile, pulmonary involvement was seen in two cases (interstitial lung disease and recurrent pneumonia). Proliferative lupus nephritis was found in kidney biopsy in 48.6%. Four cases had end stage kidney disease and had hemodialysis sessions. Management consisted of hydroxychloroquine at 5 mg/kg/day and oral steroids at a starting dose of 2 mg/kg/day in all cases, while pulses of methylprednisolone were prescribed in 57%. Other immunomodulatory treatments used were IV cyclophosphamide in 34.4%, mycophenolate mofetil in 51.4%, azathioprine in 8.6%, tacrolimus in 5.7%, rituximab in 11.4%, and methotrexate in 2.8%. Evolution was marked by stabilization in 42.8% and recurrent flares in 28.6%. Meanwhile six cases were lost to follow-up and four cases died. The reasons for mortality were severe disease activity (seen in two cases), infection and macrophage activation syndrome (in one case each).

**Conclusion:**

Management of juvenile SLE can be challenging especially when there is severe systemic involvement. Disease damage, side effects of drugs and frequent hospitalization have major effect on the quality of life of pediatric patients.

